# Emergency medical dispatcher training as a strategy to improve pre-hospital care in low- and middle-income countries: the case study of Nepal

**DOI:** 10.1186/s12245-021-00355-8

**Published:** 2021-05-06

**Authors:** Claire L. Jacobson, Samjhana Basnet, Ajay Bhatt, Shreejesh Parajuli, Sanu Krishna Shrestha

**Affiliations:** 1 Stanford University School of Medicine, Stanford, CA 94305 USA; 2 Dhulikhel Hospital, Kathmandu University, Dhulikhel, 45200 Nepal; 3 University of Hawaii, John A. Burns School of Medicine, 651 Ilalo St, Honolulu, HI 96813 USA

**Keywords:** Pre-hospital care, Emergency Medical Dispatcher, Emergency Medical Services, Nepal

## Abstract

**Background:**

Low- and middle-income countries (LMICs) often face significant challenges related to providing effective pre-hospital care services. Barriers to providing care include lack of financial resources, poor road infrastructure, lack of trained first responders and ambulance staff, and issues regarding coordination/communication between different entities involved in Emergency Medical Services. Prior initiatives to characterize and improve the state of pre-hospital care in LMICs have largely focused on improving access to high-quality ambulance services by providing training programs to community first responders and ambulance staff on how to recognize and manage key emergency conditions. In this article, we discuss an alternative strategy for improving pre-hospital care: the creation of a context-specific Emergency Medical Dispatcher (EMD) training curriculum and program.

**Methods:**

We describe the current pre-hospital care setting in Nepal, the process of creating and piloting the Nepal-specific EMD training manual, and the early impact of its implementation.

**Results:**

The 30-h EMD training was designed, piloted, and revised in collaboration with the three largest EMS organizations in Nepal. The training is now required for all dispatchers at the Dhulikhel Hospital Dispatch Center, one of the largest ambulance dispatch networks in Nepal. Dispatchers are trained in the following knowledge and skill areas: telecommunication guidelines, triaging and documentation procedures, delivery of Basic Life Support instructions to callers, other medical and trauma-condition specific instructions, and limited resource management. The short-term positive impacts of the training’s implementation include improved documentation procedures, better prioritization of ambulance resources, delivery of Basic Life Support instructions to callers, and improved communication between dispatch, responders, and healthcare facilities.

**Conclusions:**

Context-specific Emergency Medical Dispatch training programs, which aim to optimize the emergency resources available in resource-limited settings, present a promising low-cost, high-impact interventional strategy to strengthen the pre-hospital care systems in low- and middle-income countries.

## Background

Famous for its Himalayan views and cultural diversity, Nepal, like many other low- and middle-income countries (LMICs), has inadequate Emergency Medical Services. In a country of approximately 30 million people, there exist fewer than 30 total equipped ambulances with a trained healthcare professional (paramedic, EMT, or nurse) on board [1]. The more than 200 remaining “ambulance” vehicles—owned by Nepal Red Cross Society (NRCS), privately, or by local governments—are usually equipped with only an oxygen tank and a driver who may or may not have received basic first-aid training [2].

Owing to the lack of staffed/equipped ambulances and the high cost of ambulance services, the majority of patients arrive at the Emergency Department of tertiary care hospitals in a private vehicle, taxi, or local bus [3]. Nepal’s pre-hospital care context shares many similarities with that of other LMICs; however, it is unique in that in the last decade it has witnessed the emergence of several promising organizations and institutions dedicated to providing pre-hospital care services such as Dhulikhel Hospital Emergency Medical Services (DEMS) and Nepal Ambulance Service.

Nepal Ambulance Service (NAS), the first official pre-hospital care organization in the country, is a non-profit organization that currently owns and operates nine ambulances, six of which are located in Kathmandu Valley (population ~ 7 million), and three of which are in other cities in different regions [4]. NAS offers a 3-month Emergency Medical Technician (EMT) training course for paramedics (Community Medical Assistants or Health Assistants) and nurses to specialize in pre-hospital care [4, 5]. NAS provided crucial ambulance services during the 2015 earthquakes in Nepal which killed almost 9000 and injured over 22,000 people [6].

Then, in 2013, Dhulikhel Hospital, Kathmandu University’s non-profit teaching hospital, began its own pre-hospital care service called Dhulikhel Hospital Emergency Medical Services (DEMS) [7]. DEMS, based in Dhulikhel—a small city (population 32,000) 30 km east of Kathmandu—aimed to bring pre-hospital care services to patients outside of Kathmandu Valley, serving the predominantly rural Kavrepalanchowk and Sindhupalchowk districts. DEMS, with the support of the Canadian and Nepal Red Cross Society, built a dispatch center in 2019, and now coordinates 31 ambulances located throughout the two districts. Dhulikhel Hospital owns only two of the ambulances in its system, and the others are coordinated by the DEMS Dispatch Center but owned and maintained by local municipality governments. This system of local government ambulance ownership and Dhulikhel Hospital dispatch coordination is part of the Canadian and Nepal Red Cross Society initiative to strengthen rural health. Hence, DEMS can expand without significant additional costs as more local municipality governments begin to see the positive impacts of pre-hospital care services.

Both NAS and DEMS dispatch centers can be reached by dialing Nepal’s national emergency hotline number, 102, which will connect the caller to a dispatch center with 24-h call takers. Outside of NAS and DEMS, a handful of private hospitals within Kathmandu Valley also operate one to three privately staffed/equipped ambulances, and in the case of Nepal Mediciti Hospital and Grande International Hospital even facilitate helicopter emergency transport, however, these private hospitals all operate independently [4]. As EMS organizations continue to emerge and expand without a centralized system, the coordination of different resources presents a significant challenge. Not only are there hundreds of different ambulance phone numbers (usually the ambulance driver’s personal mobile phone) that callers must dial to request ambulance ambulances, but also there is also no formal communication system between EMS and the receiving facility or additional emergency resources such as police and fire departments [4].

Despite the overwhelming challenge in Nepal of coordination between the decentralized resources that comprise an Emergency Medical System, there have been no known prior initiatives to improve coordination of pre-hospital care resources outside of an individual organization or institution. This has significant negative consequences for patients, many of whom, due to a lack of communication, are transported to a facility that does not have the appropriate resources to provide the medical service(s) they require [4]. Hence, leaders at DEMS, NAS, and NRCS agreed that focusing on improving the coordination of resources and bridging some of the pre-hospital care gaps in a resource-limited, geographically and culturally diverse country like Nepal could greatly improve the state of Nepal’s Emergency Medical Services system. To address several of these important issues, leaders from DEMS, NAS, and NRCS worked together to help create Nepal’s first Emergency Medical Dispatcher (EMD) manual and training materials. The manual was created to improve the coordination, expediency, and efficacy of emergency medical transport by training an Emergency Medical Dispatcher—the key person in charge of organizing and dispatching EMS resources—in the most effective ways to connect the patient to EMS resources.

The concept of creating context-specific dispatching training programs has been discussed in several articles and studies written about pre-hospital care in LMIC contexts [5, 8]. Neil Thomson, in his study about the state of EMS in Zimbabwe, wrote, “Brand-name systems such as the Advanced Medical Priority Dispatch System (AMPDS) are too expensive to use fully in Zimbabwe, so services develop their own priorities and pre-arrival instructions (if any). This is clearly an area that needs to be developed in the future” [8]. As each dispatcher responds to, on average, hundreds to even thousands (depending on the organization) of calls every year, interventional programs involving dispatcher training are a low-cost, high-impact strategy for strengthening pre-hospital care in LMIC contexts. To date, however, few articles have explored how such an intervention would be designed and implemented. While our EMD training program was designed to work best in the Nepal pre-hospital care context, the process, challenges, and benefits of its implementation including designing the curriculum, collaborating with community partners, piloting the training, and planning its expanded integration can be applied to other LMICs working to develop their own EMD training program.

## Methods

### Training material development

The standardized dispatch protocols employed in most high-income countries are designed for a well-integrated network of pre-hospital care resources where communication through a dispatching software system is available. Using standardized international dispatch systems such as AMPDS was neither financially sustainable nor contextually appropriate given the vast differences in EMS infrastructure and limited, decentralized pre-hospital care resources available in Nepal [5, 9]. Hence, a team of Nepalese and international Emergency Medicine physicians, dispatchers, paramedics, EMTs, and healthcare professionals from the Nepal and Canadian Red Cross Society came together to create training materials and assessment measures specifically for the Nepal EMD program. The team of collaborators examined several international dispatch system models for guidance on the overall content structure and generalizable medical knowledge/skill areas to include, but the majority of the content had to be recontextualized or newly created to adequately fill the needs of Nepal’s prehospital care system. For example, the Nepal training materials had to include dispatch protocols and pre-arrival instructions for situations such as a caller reporting a landslide-induced bus accident with more than 75 victims and only two staffed ambulances owned by different organizations within a 30-min radius—an unfortunately common occurrence in the hilly regions of Nepal.

Additionally, the training was designed to be appropriate for both Emergency Medical Dispatchers working in one of the two dispatch centers in Nepal or for ambulance staff (paramedics/EMTs/first responders) who might receive calls directly from an individual in his/her community. This was a necessary component of the training curriculum as often community members prefer to directly call the phone number of a known ambulance staff member—either because they do not know the dispatch center number or because they think it will be faster to call the ambulance directly—and so the ambulance staff needs to be prepared to answer emergency phone calls, get necessary information, give pre-arrival instructions, and communicate with dispatch.

### Training content

Table 1 shows the table of contents for the topics included in the most recent version of the Emergency Medical Dispatcher curriculum. Topics covered include telecommunication guidelines, triaging, documentation procedures, Basic Life Support instructions, tertiary care hospital information, and protocols for 25 specific other medical/trauma situations. The training takes about 30 h excluding breaks and is designed to be completed in four days. We advise that the ratio of trainees to instructors should be no more than 4:1 owing to the scenario-based interactive learning structure of the training.

**Table 1:** EMD training curriculum content

Topic	Subtopics/description
**Chapter 1:** Telecommunications Guidelines	● Phone interview guidelines for primary patients ● Healthcare facility referral communication process ● Prank callers and other non-emergency calls ● Connecting callers to additional resources
**Chapter 2:** Triaging and Documentation Procedures	● Triage protocols and criteria (red, orange, yellow, green, black) ● Mass casualty incident triage and response ● Documentation procedures
**Chapter 3:** Basic Life Support Instruction Scripts	● AED instructions ● CPR adult instructions ● CPR child instructions ● CPR infant instructions ● CPR neonate instructions ● Choking child/adult instructions ● Choking infant instructions ● Airway control instructions ● Childbirth instructions
**Chapter 4:** Other Conditions: (background information, signs/symptoms, additional questions to ask, and pre-arrival instructions for patient)	● Abdominal/back/groin pain ● Anaphylaxis/allergic reaction ● Animal bites ● Assault/trauma ● Bleeding non-traumatic ● Breathing difficulty ● Burns ● Cardiac arrest ● Chest pain ● Choking ● Diabetic emergency (hyperglycemia/hypoglycemia) ● Drowning/water-related injury ● Environmental (hypothermia/hyperthermia) ● Falls/accidents/pain ● Head/neck ● Infectious disease ● Mental/emotional/psychological ● Overdose/poisoning ● Pediatric emergencies ● Pregnancy/childbirth/gynecology ● Road traffic accidents ● Seizures ● Sick (unknown)/other ● Stroke (CVA) ● Unconscious/unresponsive
**Chapter 5:** Practice Scenarios	● Over 25 practice scenarios with example scripts for trainers to practice with participants
**Chapter 6:** Resources	● List of specialty treatment hospitals ● Contact information for tertiary care hospitals

The full curriculum is available in both Nepali, the official language of Nepal, and English. The team of creators of the dispatch manual developed and selected the content based on needs identified by Nepalese EMS leaders from Dhulikhel Hospital, Nepal Ambulance Services, and Nepal Red Cross Society as well as from pre-existing, evidence-based international dispatcher guidelines such as the Criteria Based Dispatch system developed by King County Emergency Medical Services Division, USA. Whenever possible, the content was designed to match existing protocols and systems in place in Nepal. For example, in the Emergency Department of Dhulikhel Hospital, the triage levels are red (immediate medical attention needed), orange (within 20 min), yellow (within 1 h), green (within 4 h), and black (deceased). To minimize potential confusion for the dispatchers who also often serve as paramedics in the Emergency Department, we decided to maintain this same triage system to minimize potential confusion.

Currently, the only software utilized in dispatch centers is a GPS-tracking software so that dispatchers can track ambulance location and movement. None of the EMS systems in Nepal have a dispatching software or radio system that allows them to communicate with responders or hospitals using any method other than phone calls. As a result, all information must be collected and communicated through phone calls between the dispatcher and callers, responders, and hospital staff. Hence, the dispatcher curriculum focused primarily on how to quickly mobilize resources and efficiently gather and share crucial information about a patient’s condition.

We designed a scenario-based skills assessment to test dispatchers’ knowledge before (pre-test) and after (post-test) the training. Each dispatcher was evaluated individually by the same instructor using a pre-designed checklist of 10 items to evaluate whether the dispatcher correctly gathered the correct information from the caller, asked condition-specific follow-up questions, gave appropriate pre-arrival instructions, documented the call, dispatched the correct resources in the correct order, and maintained professional etiquette throughout. The same actor who played the role of the caller was used for each assessment. A score of 80% was determined to be the minimum passing score. The dispatcher would be offered a retest if he/she scored below 80%

After the initial pilot of the training, we added a second “take-home” portion of the post-training assessment, a written assessment that included eight different “mini-scenarios” where the dispatcher is given a one or two sentence description of an emergency from a hypothetical caller (i.e., “There has been a major accident here on the highway. Lots of people are hurt!”). In short answer form, the dispatch is then asked to list how they would respond including (1) what first questions they would ask the caller, (2) steps needed to dispatch the correct resources, (3) instructions given to the caller, and (4) information they would provide to the responding ambulance and/or hospital staff. The short answer responses were reviewed by the lead instructor and any incorrect or concerning responses were addressed individually with the dispatcher and discussed among the group. Completion of the written portion and an 80% or greater score on the skills examination were required to become a certified emergency medical dispatcher.

In February 2020, the academic review committee of Dhulikhel Hospital, Kathmandu University officially endorsed our training. In February 2021, we sought government endorsement from Nepal’s National Health Training Committee as part of the effort to combine private and government national efforts to strengthen pre-hospital care. We are now in the process of finalizing this endorsement to establish this as the national dispatcher training curriculum of Nepal.

## Results

### Training pilot and implementation

In October 2019, we conducted two pilot trainings of the Emergency Medical Dispatcher Curriculum at Dhulikhel Hospital with two groups of dispatchers (four participants in the first group, three in the second) from Dhulikhel Hospital and Nepal Red Cross Society. All of the dispatchers had more than one year of experience as a dispatcher but had never received any formal training on how to dispatch ambulances. We obtained informed consent from all training participants prior to beginning the training. Ethical approval for the intervention was obtained from the Institutional Review Committee at Kathmandu University.

In the pilot training implementation at Dhulikhel Hospital, the pre-test skills examination revealed that none of the seven dispatchers scored above a 3 out of 10. While all of the participants were trained paramedics, none had received training on how to dispatch ambulances, and so they were accustomed to only asking for the caller’s name, phone number, location, and sometimes, the chief complaint before disconnecting and sending an ambulance. After the training, all seven dispatchers achieved the passing score of 80% or greater on the scenario-based skills evaluation and only one participant required a re-test. Post-training, we also collected anonymous feedback forms in which we received feedback about the content of the training, the instructors, the schedule, and the overall quality of the training. Additionally, for one month following the training, we gathered weekly data through online surveys from all 7 dispatchers to assess their level of satisfaction with the dispatch protocols, challenges with using the protocols, and additional feedback about the dispatch protocols. In addition, we reviewed the Dhulikhel Hospital Dispatch Center records to assess how well the training protocols had been implemented and for changes in the outcomes associated with the dispatcher training which included: ambulance dispatch time, triaging, information gathered from the caller, instructions given to the callers and responders, communication with receiving hospitals, and documentation procedures.

Using the feedback forms, survey data, and dispatch records, we made significant changes to the curriculum over the next few months. Changes included adding guidelines for dealing with prank callers, how to contact fire and police response units, and information about selected facilities/services available at tertiary care hospitals (catheterization lab, ICU capacity, CT scan) and their emergency department contact phone numbers. We also added the second written assessment portion of the post-training evaluation to allow for additional opportunities to discuss complicated scenarios. The additions and revisions made after the first training reflected the decentralized nature of EMS in Nepal and aimed to bridge communication gaps between previously disconnected branches of pre-hospital care.

We saw that the implementation of the dispatcher training at Dhulikhel Hospital’s dispatch center had several immediate positive implications for the Dhulikhel Hospital EMS system. The first is that dispatchers, in adherence with the protocols taught in the training, began documenting the details and outcomes of each call in their electronic medical records system. Documentation records include the call received time, ambulance dispatch time, ambulance arrival time, chief complaint, patient triage level, and the instructions given by the dispatcher, none of which were previously consistently documented. This is a significant impact of the training as it provides Dhulikhel Hospital’s dispatch center with reliable documentation about the ambulance dispatch process which can be used for quality improvement purposes and to increase accountability for the dispatchers. From the documentation, we saw that dispatchers were triaging patients, providing information about the patient to responders, contacting the emergency department to inform them of the patient’s estimated arrival time, and closing gaps in the previously disconnected links in the EMS system.

The training also improved the ambulance resource allocation. Previously, dispatchers would immediately send an ambulance to a caller on a first-come, first-served basis, but by enacting the dispatching triage system and calling or providing alternative ambulance service phone numbers to lower priority patients, the dispatch center can better coordinate care for the most acute patients. Lastly, in the three months following the training, two of the seven initially trained dispatchers reported providing callers with BLS instructions which the caller was able to follow until the ambulance arrived. One of the dispatchers also reported providing BLS instructions to a newly trained paramedic who was on one of the ambulances. Given that ambulances in the hilly region of Nepal often take more than 30 min to arrive, the dispatchers’ new responsibilities to ask about a patient’s condition, gather critical information, provide instructions, and coordinate between the caller, the responding unit(s), and the hospital all mark a significant improvement in the overall EMS system. We are now monitoring and evaluating the long-term effects of the EMD training on the efficiency and quality of the EMS system.

Next, after review and approval from Dhulikhel Hospital, NRCS, and NAS, we planned for a multiorganizational dispatcher training involving 20 dispatchers from DEMS, NRCS, and NAS to occur the week of April 17, 2020. Due to the COVID-19 pandemic, however, we were not able to safely conduct the larger-scale training as we anticipated. Instead, each organization individually conducted the dispatcher training program in small groups to maintain social distancing. Until the threat of COVID-19 is under control and it is safe and possible to implement an annual week-long, joint training between all organizations, each organization is continuing to train new dispatchers using the Emergency Medical Dispatcher Curriculum for Nepal.

### Limitations

While the dispatcher training program aims to improve pre-hospital care communication and coordination, there are several features of Nepal’s pre-hospital care system that limit the expediency of the emergency dispatch system. The first is the lack of a centralized three-digit toll-free phone number used by all Emergency Medical Services. While NAS and DEMS ambulances can be activated by dialing 102, the majority of ambulance vehicles (especially in rural areas) are not connected to either dispatch network and can only be called via a specific phone number. Public awareness of these alternative ambulance service phone numbers is limited. Hence, there are often multiple calls needed to activate ambulance services, which causes unnecessary delays. Additionally, it is very difficult to obtain the appropriate emergency phone numbers of tertiary care hospitals and ambulance services. Most of these phone numbers were not available online and had to be obtained through talking to local governments or going in-person to tertiary care hospitals in Kathmandu Valley. Since many of these phone numbers are individuals’ mobile phone numbers, they are not reliably staffed and are subject to change. Secondly, owing to heavy restrictions on the use of ultra-high frequency radio use in Nepal, all pre-hospital communication must occur via cell phone. Nepal is one of the top ten least urbanized countries in the world, and outside of major cities, cell phone service is often unreliable [10]. Hence, communication between dispatcher and ambulance staff or ambulance staff and the hospital is often disrupted or delayed.

Lastly, the issue of locating patients is prevalent in the Nepal context. Triangulation of patient location using cell phone signaling is not yet possible in either dispatch center in Nepal, so the patient location must be manually communicated to the ambulance staff by the dispatcher. Most homes and buildings do not have an official address and so it can often be difficult to find the patient, especially if the area is unfamiliar to the driver and ambulance attendant. As a result, it is crucial that the ambulance staff have extensive knowledge and experience of the area that they are stationed in because directions are often given by the caller in terms of local landmarks. The coordination challenges present in Nepal and other LMIC contexts are often outside of the control of pre-hospital care organizations/institutions and emphasize the importance of working closely with government officials on pre-hospital care initiatives.

### Future directions

The next steps for the dispatcher training program are to finalize the National Health Training Center’s endorsement for the training and to develop and gain endorsement for an official Trainer of Trainers (ToT) program to certify EMD instructors. We also recognize that recertification trainings are a necessary part of continuous learning and are in discussion about the frequency and style of “refresher” trainings for dispatchers who have already completed the training once. It was decided that, once a year before the dispatcher training, previously certified dispatchers will be evaluated using the same scenario-based simulation and checklist as the training pre- and post-test evaluation, and any dispatcher who fails to meet the 80% passing threshold within three attempts will be asked to attend the training again. Once the number of dispatchers increases and Nepal’s EMS infrastructure has expanded, these requirements will likely need to be modified. Additionally, we aim to design and implement a low-cost dispatching communication software system that will address the communication gaps and delays caused by not having reliable communication means between dispatchers, callers, and responders. We hope that this dispatching software will eventually be able to scale to other LMICs in similar situations.

Owing to the multitude of recent initiatives to improve emergency care in Nepal, the landscape of ambulance services and emergency medicine is ever-changing, especially in districts near Kathmandu such as Kavrepalanchowk, Sindhupalchowk, and Sindhuli. The Emergency Medical Dispatcher curriculum and training program will have to adapt to those changes as a result. Future initiatives should focus on optimizing the present resources (i.e., centralizing all ambulances to the three-digit emergency phone number to minimize resources spent on ambulance dispatch) in addition to expanding the number of appropriately staffed and equipped ambulances. To do this, there need to be more opportunities for Nepal-specific training programs for ambulance attendants (paramedics, EMTs, nurses, and first responders) and ambulance drivers. Lastly, owing to the lack of centralized financial resources to allocate towards pre-hospital care, there needs to be a greater emphasis and research on the Nepal Red Cross Society model of municipality-level government ownership/sponsorship of ambulances to distribute the cost of upgrading ambulances with advanced medical equipment and a trained attendant. The three leading EMS organizations should also seek additional financial and infrastructural support from the national government, as the government’s support is crucial in assisting in the maintenance and expansion of these organizations.

## Conclusions

We created and implemented a Nepal-specific Emergency Medical Dispatcher training curriculum in the three largest EMS organizations in Nepal. The EMD training program works to centralize resources and mitigate the communication gaps present in a low-resource prehospital care context. The implementation of context-specific EMD Training programs is an innovative, cost- and resource-efficient strategy for strengthening pre-hospital care in low- and middle-income countries.

## Data Availability

Not applicable

## Abbreviations

EMS: Emergency medical services
EMD: Emergency medical dispatcher
DEMS: Dhulikhel hospital emergency medical services
NAS: Nepal ambulance service
NCRS: Nepal red cross society
